# Nonlinear expansion, spatial diffusion, and climate-driven dynamics of scorpionism in Brazil: a nationwide time-series and DLNM analysis (2007–2025)

**DOI:** 10.1007/s00484-026-03311-4

**Published:** 2026-08-31

**Authors:** Elania Barros da Silva, José Francisco de Oliveira Júnior, Amaury de Souza, David Mendes, Mônica Dayane Albuquerque Tenório, Luis Felipe Francisco Ferreira da Silva, Kelvy Rosalvo Alencar Cardoso, Sudhir Kumar Singh

**Affiliations:** 1 Postgraduate Program in Biosystems Engineering (PGEB), Federal Fluminense University (UFF), Niterói, Rio de Janeiro 24210-240 Brazil; 2 Institute of Atmospheric Sciences (ICAT), Federal University of Alagoas, Maceió, Alagoas 57072-260 Brazil; 3 Federal University of Mato Grosso do Sul, C.P. 549, Campo Grande, MS 79070- 900 Brazil; 4 Post-Graduate Program in Climate Sciences (PPGCC), Federal University of Rio Grande Do Norte (UFRN), Campus Universitário Lagoa Nova, Natal, Brazil; 5 Postgraduate Program in Biometrics and Applied Statistics (PPGBEA), Federal Rural University of Pernambuco (UFRPE), Recife, Pernambuco 52171-900 Brazil; 6 Department of Plants, Soils and Climate, Utah State University (USU), 4820 Old Main Hill, Logan, UT 84322 USA; 7 K. Banerjee Centre of Atmospheric & Ocean Studies (KBCAOS), Nehru Science Centre, University of Allahabad, Prayagraj, Uttar Pradesh India

**Keywords:** Scorpion sting incidence, Climate drivers, Non-linear modeling, Lag effects, Environmental epidemiology

## Abstract

Scorpionism has emerged as a growing public health concern in Brazil, characterized by increasing incidence and geographic expansion. Despite this trend, studies integrating climatic drivers and advanced time-series modeling remain limited. This study aims to analyze the temporal dynamics, spatial diffusion, and climate–disease relationships of scorpion sting incidence in Brazil using a nationwide dataset from 2007 to 2025. Monthly scorpion sting data from SINAN/DATASUS were combined with high-resolution climatic variables from the TerraClimate dataset. Descriptive statistics, seasonal analysis, and lagged correlation were applied to explore temporal patterns. A distributed lag non-linear model (DLNM) was used to quantify the non-linear and delayed effects of temperature and precipitation on scorpion sting incidence. The results revealed a pronounced non-linear increase in scorpionism, with a structural breakpoint around 2011 indicating accelerated expansion. Seasonal patterns showed higher incidence between September and November. Lag analysis demonstrated that temperature had the strongest association with incidence, peaking at a one-month lag (*r* ≈ 0.57), while precipitation exhibited weaker but delayed effects. DLNM results confirmed significant non-linear and lag-dependent relationships, with elevated temperatures associated with increased risk at short lag periods. Scorpionism in Brazil is a climate-sensitive and dynamically evolving epidemiological system, driven by the interaction of environmental variability and urban expansion. These findings highlight the need for integrated, climate-informed public health strategies and predictive modeling approaches to mitigate future risk.

**Supplementary Information:**

The online version contains supplementary material available at https://doi.org/10.1007/s00484-026-03311-4.

## Introduction

Scorpionism represents an increasingly important public health issue in tropical and subtropical regions, particularly in Latin America, where environmental conditions and urbanization processes favor the proliferation of synanthropic scorpion species (Torrez et al., 2019; Guerra-Duarte et al., 2023). It is worth noting that factors such as social inequality, the degradation of environmental quality in urban areas, and inadequate waste management favor the proliferation of scorpions (Almeida et al., 2021; Silva Pinheiro et al., 2026). In Brazil, scorpion sting accidents have shown a consistent increase over the past decades, becoming one of the most relevant causes of envenomation, with significant implications for morbidity and healthcare systems (Reckziegel & Pinto, 2014; Borges et al., 2025).

The expansion of scorpionism has been strongly associated with rapid and often unplanned urbanization, inadequate sanitation infrastructure, and the remarkable ecological plasticity of species such as *Tityus serrulatus*, which are highly adapted to urban environments (Zanetta et al., 2020; Lacerda et al., 2022; Pucca et al., 2025). These factors create favorable conditions for scorpion survival, reproduction, and increased human–scorpion interaction.

In addition to anthropogenic drivers, environmental and climatic factors are increasingly recognized as key determinants of scorpion activity and distribution. Temperature, precipitation, and humidity influence metabolic rates, reproductive cycles, shelter availability, and prey dynamics, thereby affecting the risk of scorpion sting incidents (Lourenço, 2018; Guerra-Duarte et al., 2023) Recently, several studies have suggested that climate change, together with land-use and land-cover changes, may further intensify the risk of scorpion sting occurrences (Andrade Moura et al., 2025; Karmaoui & Sereno, 2026). However, the temporal relationship between climatic variability and scorpionism remains insufficiently explored, particularly in terms of non-linear responses and delayed effects (Chiaravalloti-Neto et al., 2023; Karmaoui & Sereno, 2026).

Although previous studies have investigated temporal trends and spatial patterns of scorpionism in Brazil, most analyses rely on descriptive approaches or linear models, which may not adequately capture the complexity of climate–disease interactions (Reckziegel & Pinto, 2014; Lacerda et al., 2022; Borges et al., 2025; Pucca et al., 2025). Advanced modeling frameworks, such as distributed lag non-linear models (DLNM), have been widely applied in environmental epidemiology to assess delayed and non-linear effects of climatic variables, for example, Quijal-Zamorano et al. (2024) applied these models to investigate the relationship between air temperature and mortality across the 73 neighborhoods of Barcelona, Spain, while Briz-Redón et al. (2026) used such models to establish the effects of air pollution and extreme temperatures on human health in Belgium, yet their application to scorpionism remains scarce.

This gap limits the understanding of the mechanisms underlying the observed expansion and variability of scorpion sting incidence. Therefore, there is a clear need for integrated approaches that combine high-resolution climatic data with robust statistical modeling to better characterize the temporal dynamics and environmental drivers of scorpionism.

In this context, the present study aims to investigate the nonlinear temporal dynamics, spatial diffusion, and climate-driven behavior of scorpion sting incidence in Brazil using a nationwide time-series framework combined with distributed lag non-linear modeling.

## Materials and methods

### Study area

**Fig. 1:**
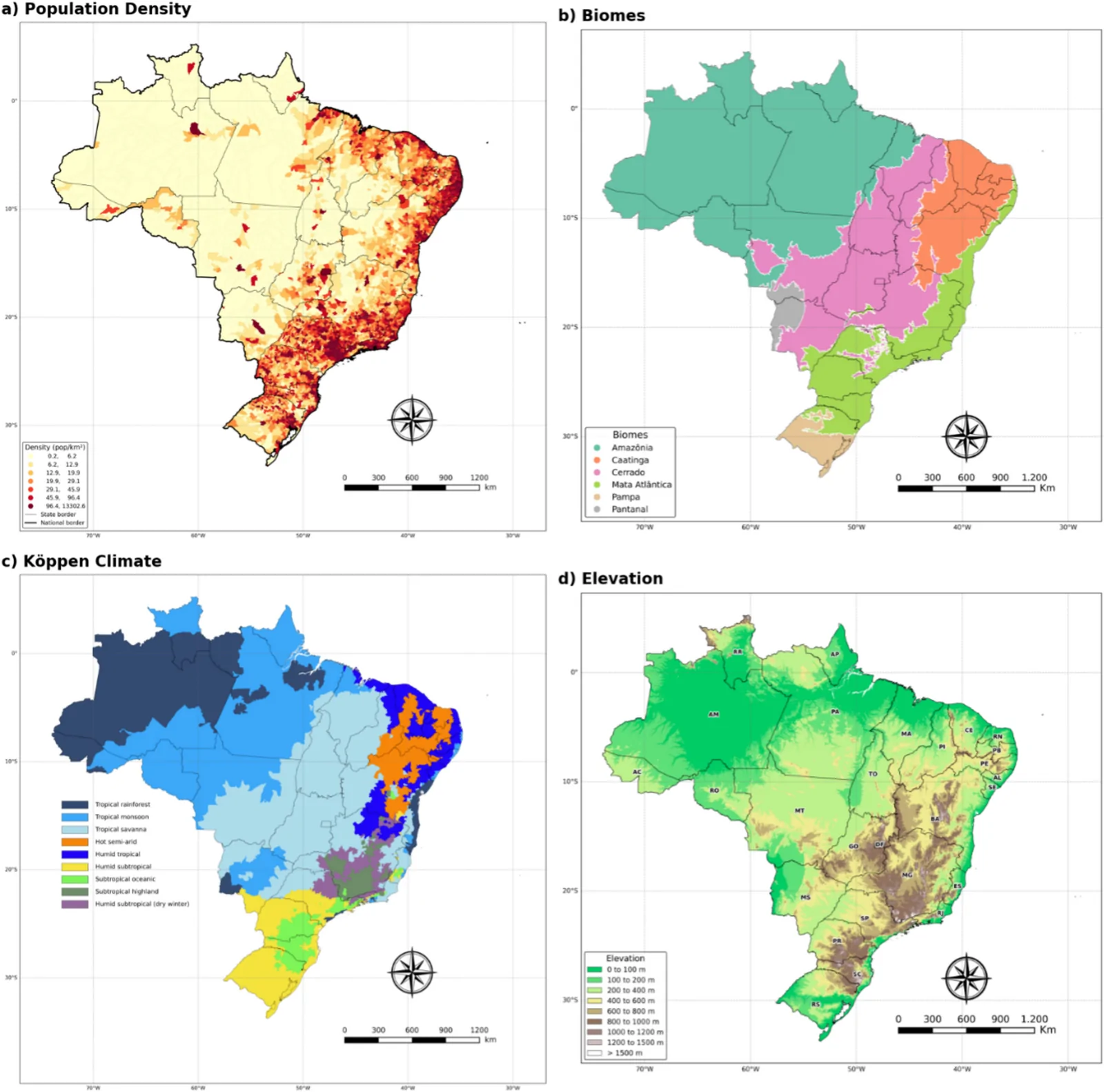
Geographic and environmental characterization of Brazil: (**a**) Population Density (pop/km^2^), (**b**) Biomes, (**c**) Köppen Climate and (**d**) Elevation (mm)

The study area corresponds to Brazil, which is located in the central-eastern portion of the South American continent, between latitudes 5°16′20″ N and 33°44′32″ S and between longitudes 34°45′54″ W and 73°59′32″ W. Brazil covers an area of 8,514,876.599 km², accounting for approximately 47% of the South American territory. Its entire northeastern (NE), eastern (E), southeastern (SE), and part of its northern coastline are bordered by the Atlantic Ocean. Currently, Brazil is geopolitically divided into 26 states and the Federal District. Population density is highest along the coast and decreases significantly toward the interior of the country (Fig. 1a). Brazil comprises six biomes: the Amazon, Caatinga, Cerrado, Atlantic Forest, Pampa, and Pantanal (Fig. 1b). The country exhibits a wide range of Köppen climate classifications, from Tropical Rainforest to Humid Subtropical (dry winter) climates (Fig. 1c). Elevation in Brazil ranges from 0 to 100 m along the coast, while inland areas present elevations varying from approximately 800 m to 1,500 m (Fig. 1d).

### Data sources

Scorpion sting incidence data were obtained from the Brazilian Notifiable Diseases Information System (SINAN/DATASUS), covering the period from 2007 to 2025. The dataset includes monthly counts of scorpion sting accidents reported across all Brazilian states, providing a comprehensive overview of the temporal evolution of scorpionism at the national scale.

Climatic data were derived from the TerraClimate dataset, a high-resolution global gridded dataset (~ 4 km spatial resolution) that provides monthly climate and water balance variables from 1958 onwards (Abatzoglou et al., 2018). For this study, climatic variables were extracted for the capital city of each Brazilian state, including maximum temperature (tmax, °C), precipitation (ppt, mm), and soil moisture (soil). These variables were selected due to their ecological relevance in influencing scorpion activity, habitat conditions, and human exposure risk.

The datasets were harmonized temporally and spatially by aggregating both epidemiological and climatic variables to a common monthly resolution. The final dataset consisted of a panel structure with observations defined by state, year, and month.

### Data preprocessing and integration

Prior to analysis, all datasets underwent a standardization process to ensure consistency and comparability. Missing values in the epidemiological data were treated as zero when explicitly reported as absent cases, while non-numeric entries were converted accordingly. Climatic variables were filtered to match the study period (2007–2025), ensuring temporal alignment with the scorpionism dataset.

A panel dataset was constructed by merging epidemiological and climatic data using state, year, and month as key identifiers. Lagged climatic variables were generated for each state using time-series shifting procedures, considering lag periods from 0 to 6 months. This approach allows for the evaluation of delayed effects of environmental drivers on scorpion sting incidence, reflecting potential ecological and behavioral response times.

### Descriptive and exploratory analysis

Because the primary objective of this study was to investigate the temporal dynamics, spatial diffusion, and climate-related variability of scorpionism in Brazil, the analyses were based on the absolute number of reported scorpion sting cases recorded in the SINAN/DATASUS surveillance system. Absolute case counts represent the official metric used by the Brazilian public health surveillance system to monitor disease burden over time and to support public health decision-making. Although incidence rates are valuable for assessing population-standardized risk, they require annual population estimates and address a different epidemiological objective. In the present study, the focus was on characterizing the evolution of the national burden of reported cases and its association with climatic variability, rather than estimating individual population risk.

Descriptive statistics were computed to characterize the central tendency and variability of scorpion sting incidence at the national scale, including mean, standard deviation (SD), maximum, minimum, and coefficient of variation (CV,%). Seasonal patterns were assessed through monthly aggregation of cases across the study period, allowing the identification of intra-annual variability.

To investigate preliminary associations between climatic variables and scorpion sting incidence, Pearson correlation coefficients were calculated for each climatic variable across multiple lag periods (0–6 months). This exploratory step provides initial insights into the temporal structure of climate–disease relationships and informs the specification of subsequent modeling approaches.

### Distributed lag non-linear model (DLNM)

To quantify the potentially non-linear and delayed effects of climatic variables on scorpion sting incidence, a distributed lag non-linear modeling (DLNM) framework was employed. DLNM allows for the simultaneous estimation of exposure–response relationships and lagged effects within a unified statistical model (Gasparrini et al., 2010; Gasparrini, 2011).

The core of the DLNM approach is the specification of a cross-basis function, which defines a bi-dimensional space describing both the functional form of the exposure–response relationship and its temporal lag structure. Natural cubic splines were used to model non-linear relationships for both the exposure and lag dimensions, with degrees of freedom selected based on model parsimony and previous applications in environmental epidemiology.

The model was specified as a generalized linear model (GLM) with a quasi-Poisson distribution to account for overdispersion commonly observed in count data. The general model structure is given by:

1 $$Cases_t\sim quasi-Poisson\left({\mu\:}_t\right)$$

    

2 $$\begin{array}{c}log\left({\mu\:}_t\right)=\alpha\:+cb\left(Temp_t,Lag\right)\\+cb\left(Precip_t,Lag\right)+s\left(Time\right)\end{array}$$

where $cb\left(\cdot\:\right)\:$represents the cross-basis function for each climatic variable, and $\:s\left(Time\right)$is a spline term used to control for long-term trends and seasonality.

Lag periods from 0 to 6 months were considered, based on ecological plausibility and exploratory lag-correlation results. Model predictions were obtained using the cross-prediction function, enabling the visualization of exposure–lag–response surfaces and the estimation of cumulative effects.

### Statistical analysis and visualization

All analyses were conducted using R software, with the DLNM framework implemented through the *dlnm* package (Gasparrini, 2011). Additional statistical procedures, including spline modeling and generalized linear models, were performed using standard R packages such as *splines* and *stats*.

Graphical outputs included exposure–lag–response surfaces, contour plots, and cumulative effect curves, which provide intuitive representations of the complex interactions between climatic variables and scorpion sting incidence. These visualizations are critical for interpreting the temporal dynamics and identifying critical lag periods associated with increased epidemiological risk.

## Results

### National temporal dynamics of scorpion sting accidents

**Fig. 2:**
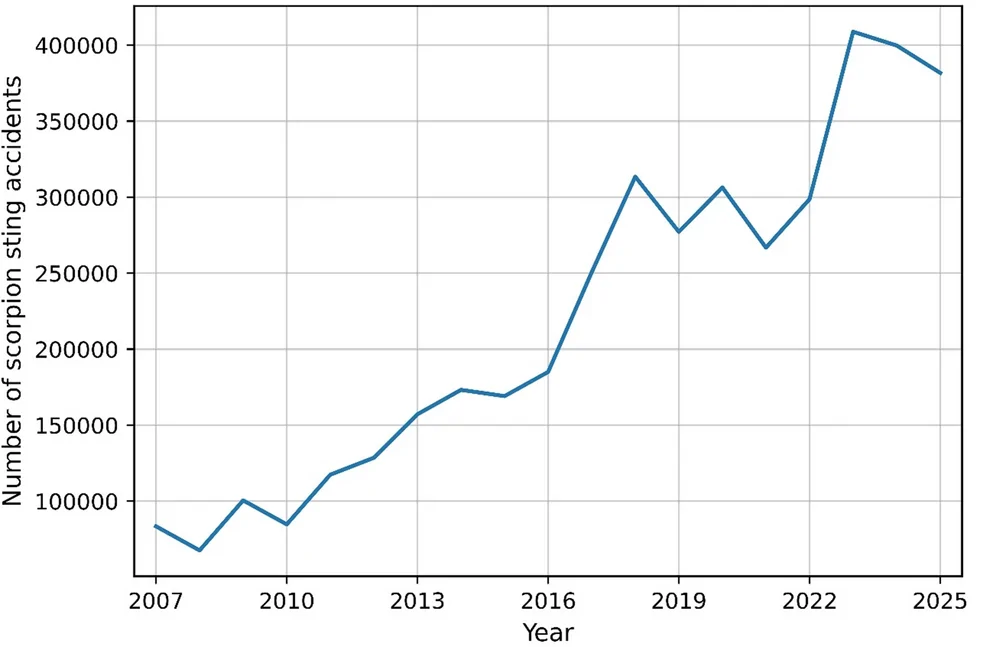
Annual number of scorpion sting accidents in Brazil (2007–2025). The time series demonstrates a non-linear increasing trend with evident interannual variability. A transition to a steeper growth regime is observed after approximately 2011, followed by a period of rapid expansion between 2017 and 2023

The national time series of scorpion sting accidents in Brazil (2007–2025) revealed a pronounced and non-linear increasing trend, characterised by substantial interannual variability and progressive acceleration over time (Table 1). Annual case counts increased from approximately 83,000 in 2007 to values exceeding 400,000 in recent years, indicating a substantial expansion of the epidemiological burden (Fig. 2). A visual inspection of the time series demonstrates a transition from moderate growth in the early period to a markedly steeper trajectory after the early 2010s, suggesting a shift in the underlying dynamics of scorpionism at the national scale.

**Table 1:** Summary statistics of scorpion sting accidents by region in Brazil from 2007 to 2025. The table presents total number of cases, mean annual cases, maximum and minimum annual values, and mean annual growth rates. Substantial regional differences are observed, with the Southeast and Northeast regions concentrating the highest number of cases, while the highest relative growth rates are observed in the South and Southeast regions

Region	Total cases	Mean annual cases	Maximum	Minimum	Mean growth (%)
Centre-West	131,347	6,913	20,989	1,132	76.85
Northeast	772,263	40,645	96,968	6,599	93.68
North	69,800	3,674	7,288	362	93.62
Southeast	863,523	45,449	96,256	1,642	113.58
South	59,903	3,153	10,166	113	115.75
Brazil (total)	2,253,902	118,626	241,133	37,332	11.47

### Growth rates and temporal instability

**Fig. 3:**
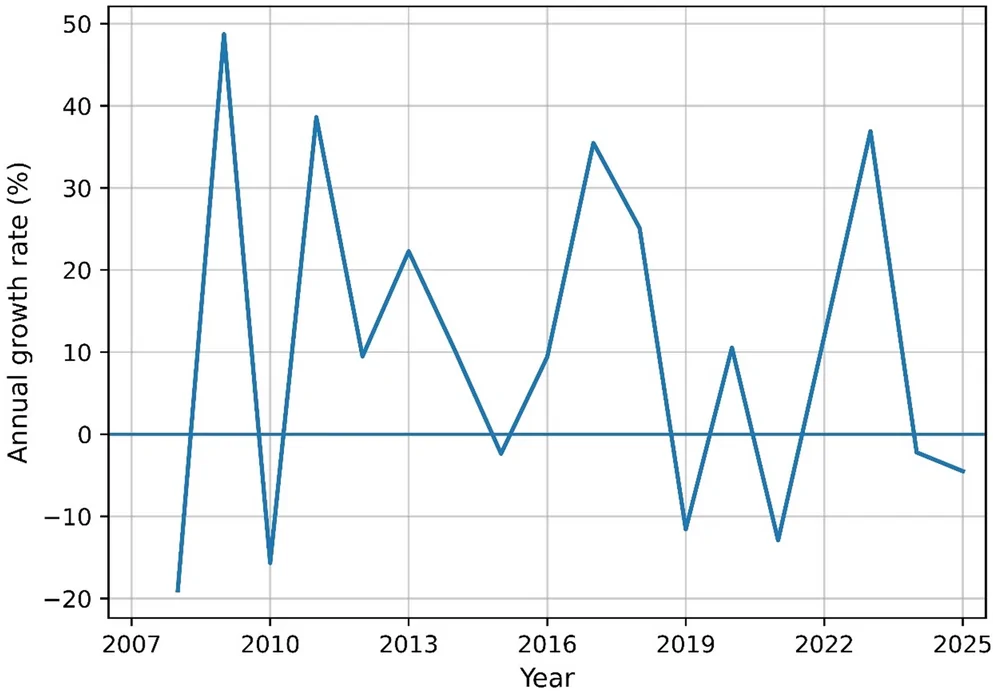
Annual growth rate (%) of scorpion sting accidents in Brazil from 2007 to 2025. The figure shows the year-to-year percentage variation in reported cases, highlighting substantial interannual variability with alternating periods of rapid expansion and temporary decline. Peaks exceeding 50% growth and negative values indicate an unstable and dynamically evolving epidemiological system, despite the overall long-term increasing trend

Annual growth rates exhibited considerable variability, ranging from negative values to peaks exceeding 50%, indicating an unstable and dynamically evolving epidemiological system. Periods of rapid expansion were interspersed with temporary declines, although these reductions were not sustained over time. Despite short-term fluctuations, the overall trajectory remained upward, suggesting that the system is characterised by persistent expansion rather than cyclical stability (Fig. 3).

### Structural change and regime transition

Breakpoint analysis identified a major structural change around 2011, separating two distinct temporal regimes. The pre-2011 period was characterised by moderate and irregular growth, whereas the post-2011 period exhibited a significantly steeper slope, indicating accelerated expansion of scorpion sting incidence.

**Fig. 4:**
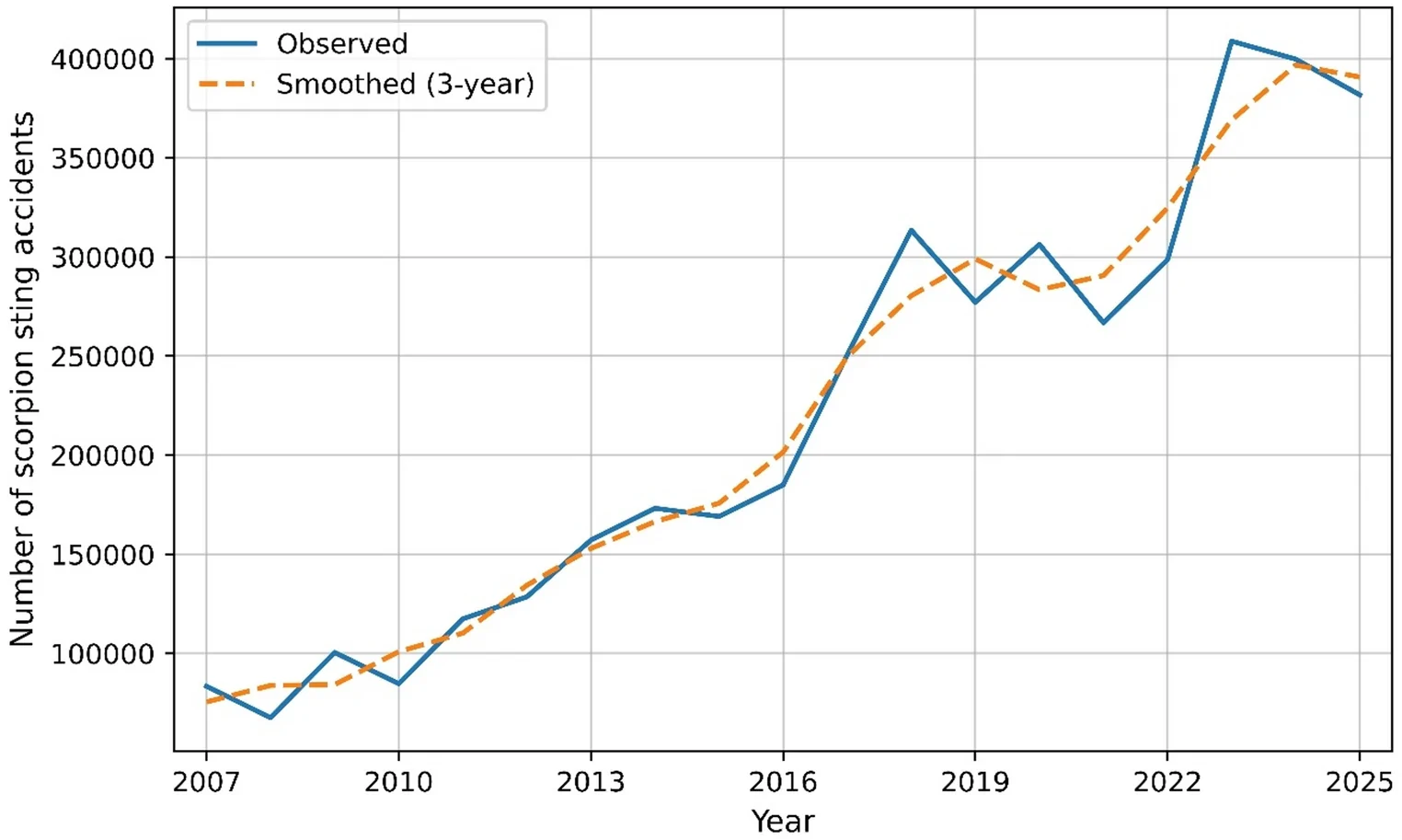
Observed and smoothed trend of scorpion sting accidents in Brazil from 2007 to 2025. The solid line represents the observed annual number of cases, while the dashed line shows the three-year centered moving average. The smoothed series highlights a persistent non-linear increase over time, with a clear acceleration after the early 2010s and no evidence of long-term stabilization despite short-term fluctuations

Smoothed trend analysis confirmed that the underlying trajectory remained consistently increasing throughout the study period, despite interannual oscillations. The most pronounced expansion occurred between 2017 and 2023, when case counts reached their highest recorded levels. Although a slight decline was observed in the most recent years (2023–2025), this reduction did not represent a structural reversal of the long-term trend but rather short-term variability within a persistently increasing system (Fig. 4).

### Regional heterogeneity in temporal patterns

**Fig. 5:**
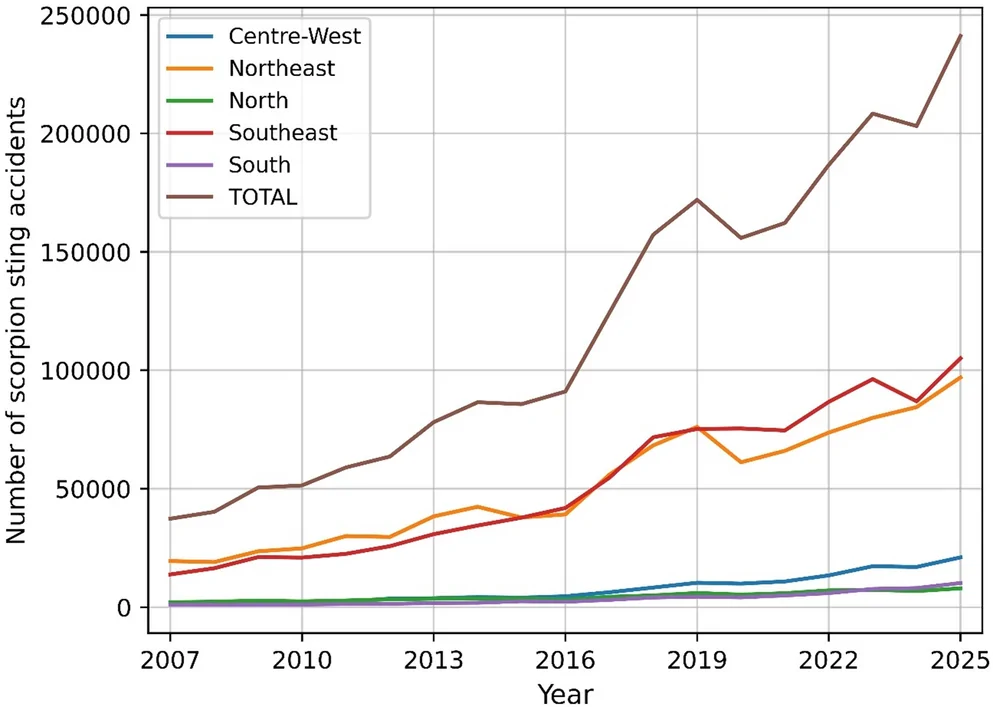
Time series of scorpion sting accidents by region in Brazil from 2007 to 2025. The figure shows marked regional heterogeneity in both magnitude and temporal dynamics. The Southeast region historically presents the highest number of cases, while the Northeast and North regions exhibit sustained expansion. The Centre-West shows delayed but accelerated growth in recent years, whereas the South maintains comparatively low and irregular incidence levels

The spatial distribution of scorpion sting accidents across Brazil (Figure MS1, Supplementary Material) reveals pronounced regional inequalities, with a well-defined concentration of cases in the Southeast and Northeast regions, while the North and South regions exhibit comparatively lower incidence levels (Fig. 5).

Substantial regional heterogeneity was observed in the temporal evolution of scorpion sting accidents across Brazil. The magnitude, growth patterns, and variability differed markedly among regions, indicating a spatially uneven expansion of the epidemiological system.

### Regional dynamics and shifting epidemiological centres

The Southeast region consistently recorded the highest absolute number of cases throughout the study period, acting as the primary historical epicentre of scorpionism. However, its trajectory showed signs of instability in recent years, including a marked decline in the most recent observations. In contrast, the Northeast region exhibited strong and sustained growth, characterised by rapid increases and high interannual variability. Temporary reductions were followed by rapid recoveries, indicating an active expansion process.

The North region showed a gradual and relatively stable increase in cases, suggesting a gradual intensification of epidemiological pressure. The Centre-West region demonstrated a delayed but increasingly accelerated growth pattern, particularly in the later years of the series, indicating a transition toward higher endemicity. The South region maintained comparatively low case numbers, with irregular fluctuations and no clear long-term increasing trend.

### Comparative regional growth patterns

Comparative analysis of regional growth rates indicated that the Southeast and South regions exhibited the highest relative increases over time, although the latter remained at low absolute levels. The Northeast and North regions also showed substantial growth, reflecting ongoing spatial expansion, while the Centre-West region displayed moderate but increasingly accelerating growth (Table 1).

### Integrated interpretation of national and regional trends

Taken together, the results demonstrate that the expansion of scorpionism in Brazil is characterised by a combination of temporal acceleration and spatial diffusion. The national trend reflects not only increasing incidence within historically affected regions but also the progressive expansion of the phenomenon into new areas. The observed patterns suggest a dynamically evolving epidemiological system with shifting centres of intensity, rather than a uniform increase across the territory.

### Climate–disease relationship

**Fig. 6:**
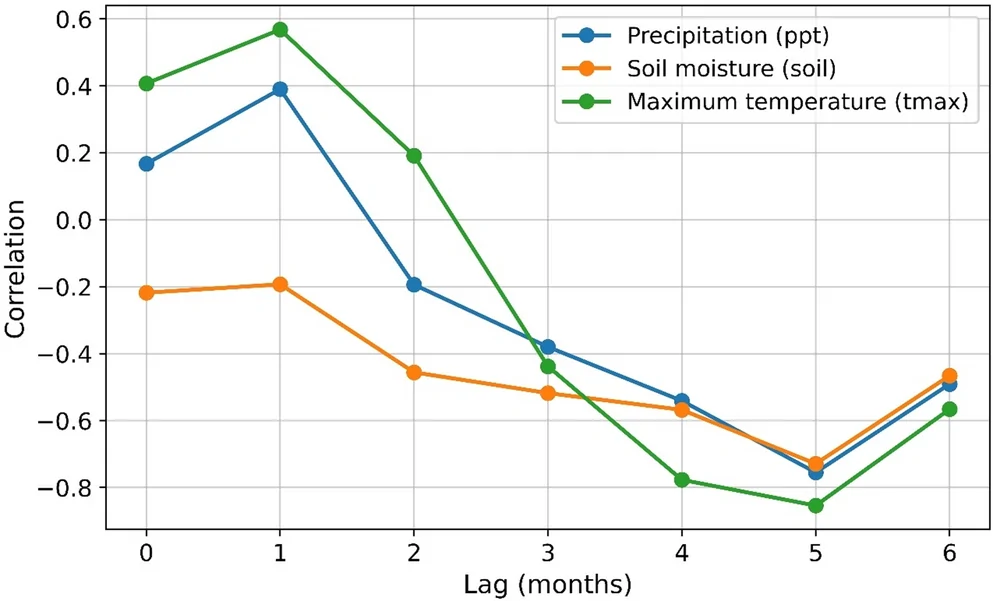
Lagged correlation between climatic variables and scorpion sting incidence in Brazil. Temperature and precipitation show peak positive correlations at a one-month lag, while soil moisture exhibits predominantly negative associations across lag periods

The lagged correlation analysis revealed a clear association between climatic variables and scorpion sting incidence across Brazilian states. Temperature exhibited the strongest relationship, with a peak correlation at a one-month lag (*r* ≈ 0.57), indicating that increases in temperature are followed by higher case counts in the subsequent month (Fig. 6).

Precipitation also showed a positive association, with maximum correlation observed at a one-month lag (*r* ≈ 0.39), suggesting a delayed effect of rainfall on scorpion activity and human exposure.

In contrast, soil moisture displayed predominantly negative correlations across all lag periods, indicating a more complex or indirect relationship with scorpion sting incidence.

Overall, the results indicate that climatic influences on scorpionism are temporally structured, with short-term delayed effects playing a key role in shaping the observed epidemiological dynamics.

### Non-linear lagged effects of climate variables

**Fig. 7:**
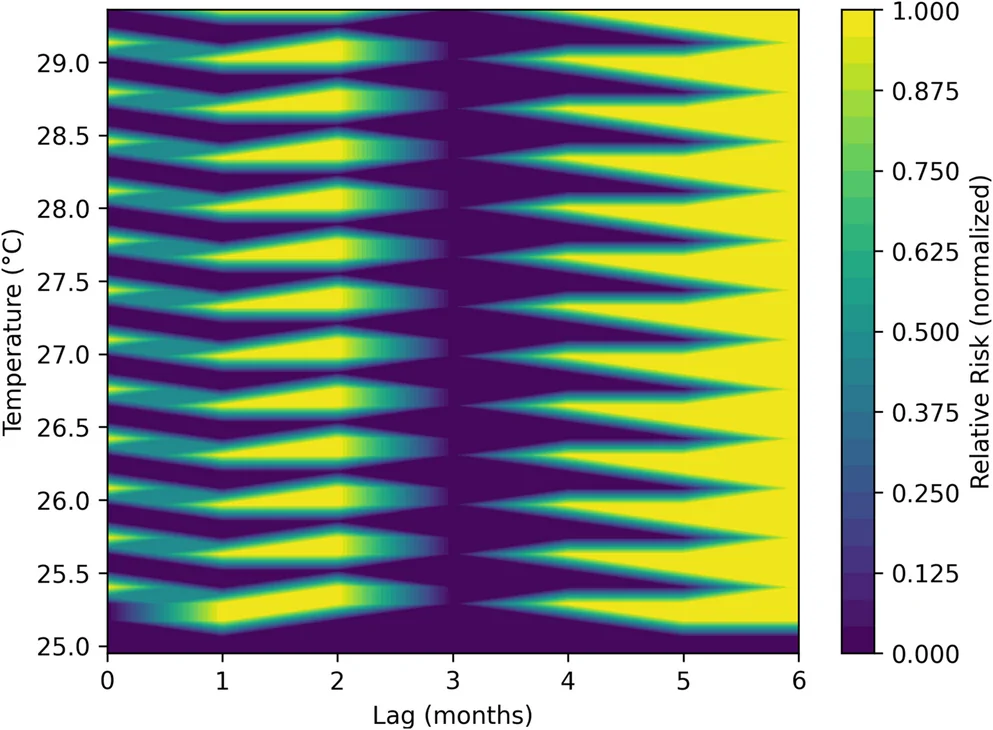
Exposure–lag–response surface showing the relationship between temperature and scorpion sting incidence. Higher temperatures are associated with increased risk at short lag periods, with effects diminishing over longer lags

The distributed lag non-linear model (DLNM) revealed significant non-linear and delayed effects of climatic variables on scorpion sting incidence. Temperature exhibited a strong positive association, with the highest risk observed at short lag periods (0–1 months), indicating a rapid response of scorpion activity to thermal conditions (Fig. 7).

The exposure–lag–response surface demonstrated that increasing temperatures were associated with elevated risk levels, although the relationship was not strictly linear, suggesting the existence of optimal thermal conditions for scorpion activity.

Precipitation also showed a significant delayed effect, with peak influence occurring at lag periods of approximately 1–2 months. This pattern suggests that rainfall may indirectly affect scorpion incidence by modifying environmental conditions such as shelter availability and prey dynamics.

Overall, the results indicate that climatic effects on scorpionism are both non-linear and temporally distributed, reinforcing the role of environmental drivers in shaping the observed epidemiological dynamics.

The exposure–lag–response surface reveals a non-linear and temporally distributed effect of temperature on scorpion sting incidence. Higher temperatures are associated with increased relative risk, particularly at short lag periods (0–1 months), indicating a rapid response of scorpion activity to thermal conditions. The effect diminishes and changes across longer lag periods, suggesting a dynamic interaction between environmental conditions and epidemiological response.

## Discussion

### A nonlinear and expanding epidemiological system

The present study demonstrates that scorpionism in Brazil is characterized by a persistent, non-linear expansion with clear evidence of temporal acceleration and structural transition. The breakpoint identified around 2011 marks a shift from moderate growth to a phase of rapid expansion, indicating a fundamental change in the epidemiological dynamics of scorpion sting incidence. These findings are consistent with previous studies that reported increasing trends in scorpionism across Brazil, particularly in urban environments (Reckziegel & Pinto, 2014; Torrez et al., 2019).

This pattern supports the interpretation of scorpionism as a self-reinforcing epidemiological system, in which ecological adaptability of synanthropic species, combined with urban infrastructure deficiencies and increased human exposure, sustains long-term growth (Zanetta et al., 2020; Lacerda et al., 2022). Rather than approaching saturation, the system continues to expand, suggesting that current control measures remain insufficient to alter its underlying trajectory.

### Temporal instability, seasonality, and climate-driven dynamics

The substantial variability observed in annual growth rates highlights the inherently unstable nature of scorpionism dynamics. Alternating phases of rapid expansion and temporary decline suggest that the system is influenced by multiple interacting drivers, including environmental variability, reporting practices, and operational responses (Lourenço, 2018; Guerra-Duarte et al., 2023).

In addition to interannual variability, the results revealed a well-defined seasonal structure. Higher incidence levels were concentrated between September and November indicate a consistent intra-annual pattern, while the CV (20–29%) suggests moderate but structured variability. This seasonal organization reinforces the interpretation that scorpionism is not governed by random fluctuations, but rather by deterministic environmental forcing combined with non-linear system dynamics (Chiaravalloti-Neto et al., 2023).

The lagged correlation analysis further demonstrated that climatic influences are temporally structured. Temperature showed the strongest association, with a peak effect at a one-month lag, indicating a rapid ecological response. Precipitation exhibited a weaker but still significant delayed effect, typically peaking at 1–2 months. These findings are consistent with ecological mechanisms in which temperature directly influences scorpion metabolism and activity, while precipitation acts indirectly by modifying habitat conditions and shelter availability (Bradley, 1988; Karmaoui & Sereno, 2026).

The DLNM results provide robust evidence that these relationships are both non-linear and lag-dependent. The exposure–lag–response surface revealed that higher temperatures are associated with increased risk, particularly at short lag periods, followed by a gradual attenuation. This behavior suggests the existence of optimal thermal conditions that enhance scorpion activity and human–scorpion encounters (Bradley, 1988; Guerra-Duarte et al., 2023). Similar patterns have been reported in studies linking environmental variability to vector- and animal-related health outcomes (Gasparrini et al., 2010; Gasparrini, 2011).

Taken together, these results support the interpretation of scorpionism as a complex adaptive system, in which climatic forcing interacts with ecological and anthropogenic factors to generate dynamic and context-dependent epidemiological patterns.

Beyond improving the understanding of climate–scorpionism relationships, the DLNM framework provides important practical applications for public health surveillance. The identification of nonlinear exposure–response relationships and short-term lag effects demonstrates that climatic information can be incorporated into operational surveillance systems to anticipate periods of increased scorpion sting risk. In particular, the approximately one-month lag observed between increases in temperature and higher scorpion sting incidence represents a valuable temporal window for preventive interventions. Climate-informed early warning systems integrating meteorological forecasts with epidemiological surveillance could therefore support proactive risk communication, optimization of antivenom distribution, targeted vector control actions, and the allocation of healthcare resources in regions with elevated vulnerability. Such predictive approaches may substantially improve preparedness and reduce the public health burden of scorpionism under ongoing climate variability and environmental change.

### Regional heterogeneity and spatial diffusion

A key contribution of this study is the identification of strong regional heterogeneity in both magnitude and growth patterns of scorpion sting incidence. The Southeast region historically functioned as the primary epicenter, concentrating the highest number of cases. However, the sustained expansion observed in the Northeast, North, and Centre-West regions indicates an ongoing process of spatial diffusion.

This shift suggests that scorpionism is no longer restricted to historically affected regions, but is progressively expanding into new areas. Such diffusion is likely driven by a combination of urban expansion, increased connectivity between regions, and the ecological plasticity of species such as *Tityus serrulatus* (Torrez et al., 2019; Lacerda et al., 2022).

Climatic variability appears to play a crucial role in modulating these spatial dynamics. Higher temperatures, precipitation regimes, and humidity conditions can directly affect scorpion reproduction, activity, and survival, while also influencing prey availability and microhabitat suitability. The observed seasonal peaks align with periods of elevated temperature and rainfall in many regions of Brazil, suggesting that climatic forcing contributes to both temporal and spatial patterns.

Additionally, large-scale climate variability, such as the El Niño–Southern Oscillation (ENSO), may influence scorpionism indirectly by altering hydroclimatic conditions across regions (Guerra-Duarte et al., 2023). These findings reinforce the idea that the spatial expansion of scorpionism is driven not only by urbanization but also by environmental and climatic drivers operating across multiple scales.

### Absence of stabilization and limitations of control strategies

Despite localized fluctuations and occasional short-term declines, there is no evidence of long-term stabilization in the national trend. The persistent increase in incidence suggests that current control strategies have limited effectiveness in addressing the structural drivers of scorpionism.

Previous studies have shown that traditional control measures, such as mechanical capture, are often reactive and reflect operational effort rather than actual reductions in scorpion populations (Lacerda et al., 2022). Even chemical control methods tend to produce localized and temporary effects, functioning more as risk modifiers than as long-term solutions.

The continued expansion observed in this study indicates that scorpionism should be understood as a systemic problem, requiring integrated and preventive strategies rather than isolated interventions.

### Implications for public health policy

The findings of this study have important implications for public health policy in Brazil. The combination of temporal acceleration, spatial diffusion, and climate sensitivity suggests that scorpionism should be reframed as a large-scale environmental and urban health challenge.

Effective mitigation strategies should include:

- integration of epidemiological surveillance with urban planning and sanitation policies.
- targeted interventions in high-risk environments (e.g., sewer systems, waste accumulation areas).
- strengthening healthcare systems for early diagnosis and treatment.
- public education campaigns aimed at reducing human exposure.

Importantly, policies should move beyond reactive approaches and incorporate predictive frameworks that account for climatic variability and environmental risk factors.

Considering the marked regional heterogeneity observed in this study, public health interventions should also be tailored to the epidemiological characteristics of each Brazilian region. In the Southeast, where the highest historical burden of scorpionism was observed, priority should be given to integrated urban pest management, sanitation improvements, and continuous surveillance. In the Northeast, where incidence continues to expand rapidly, strengthening epidemiological surveillance, community education, and early diagnosis should be prioritized. In the Centre-West and North regions, where scorpionism is undergoing progressive expansion, surveillance systems should focus on the early detection of emerging hotspots and climate-informed risk monitoring. Although the South currently presents comparatively lower incidence, preventive surveillance should be maintained because environmental and climatic changes may facilitate future geographic expansion. These region-specific strategies may improve the efficiency of public health interventions by aligning surveillance and prevention measures with the distinct epidemiological dynamics identified across Brazil.

### Limitations

This study presents some limitations that should be acknowledged. First, the use of aggregated monthly data may mask finer-scale temporal dynamics. Second, potential biases related to underreporting and variability in surveillance systems may affect the accuracy of incidence estimates. Third, although climatic variables were incorporated, other environmental and socio-economic factors were not explicitly included in the modeling framework.

Furthermore, while the DLNM approach provides strong evidence of association, causal relationships cannot be definitively established within an observational framework.

Overall, the results indicate that scorpionism in Brazil is undergoing a process of nonlinear expansion and spatial redistribution, driven by the interaction of climatic variability, urbanization, and ecological adaptability. The system operates under persistent epidemiological pressure, characterized by structured variability, delayed environmental effects, and absence of stabilization.

These findings highlight the need to conceptualize scorpionism as a complex and evolving public health issue, requiring integrated, long-term, and multidisciplinary approaches to effectively mitigate its impact.

## Conclusion

This study provides a comprehensive assessment of the temporal dynamics, spatial diffusion, and climatic drivers of scorpionism in Brazil from 2007 to 2025. The results demonstrate that scorpion sting incidence is undergoing a persistent and non-linear expansion, characterized by temporal acceleration, structural changes, and marked regional heterogeneity.

The identification of a breakpoint around the early 2010s indicates a transition toward a more rapidly expanding epidemiological regime, while the absence of long-term stabilization suggests that current control strategies have been insufficient to contain the spread of scorpionism.

In addition to long-term trends, the analysis revealed a well-defined seasonal pattern, with higher incidence levels were concentrated in late-year months, indicating that intra-annual variability is structured rather than random. The integration of climatic data further demonstrated that environmental factors play a critical role in shaping these dynamics. Temperature emerged as the primary driver, exhibiting strong and immediate effects on incidence, while precipitation showed weaker but delayed influences.

The application of distributed lag non-linear models (DLNM) confirmed that the relationship between climate and scorpion sting incidence is both non-linear and temporally distributed, reflecting complex ecological mechanisms underlying scorpion activity and human exposure. These findings support the interpretation of scorpionism as a climate-sensitive and dynamically evolving epidemiological system.

Overall, the expansion of scorpionism in Brazil reflects the combined effects of urbanization, ecological adaptability, and climatic variability, resulting in a persistent and spatially heterogeneous public health challenge.

From a policy perspective, these results highlight the need for integrated and forward-looking strategies that incorporate environmental management, urban planning, and climate-informed surveillance systems. Future research should focus on incorporating additional environmental and socio-economic variables, as well as developing predictive models capable of anticipating high-risk periods and regions.

Ultimately, addressing scorpionism in Brazil will require a shift from reactive control measures toward a preventive, systemic, and multidisciplinary approach capable of responding to the complex drivers of this emerging public health issue.

## Electronic Supplementary Material

Below is the link to the electronic supplementary material.

Supplementary Material (DOCX 144 KB)

## Data Availability

The data that support the findings of this study are available from the corresponding author upon reasonable request. The data used in this study are publicly available from the Brazilian Information System for Notifiable Diseases (SINAN/DATASUS).
