# Supplementary material for: Nonlinear expansion, spatial diffusion, and climate-driven dynamics of scorpionism in Brazil: a nationwide time-series and DLNM analysis (2007–2025)
Source: Int J Biometeorol. 2026 Aug 31;70(9):245. doi: 10.1007/s00484-026-03311-4 (PMC13529833; doi:10.1007/s00484-026-03311-4)
Supplement: Supplementary file 1 [file 484_2026_3311_MOESM1_ESM.docx]

**Supplementary Material**

**Figure MS1.** Spatial distribution cases of scorpion sting accidents in Brazil from 2007 to 2025 based on SINAN/DATASUS data.

Figure MS1 presents the spatial distribution and cumulative number of scorpion sting accidents in Brazil from 2007 to 2025, highlighting marked regional inequalities. A well-defined spatial pattern is observed, with higher concentrations of cases in the Southeast and Northeast regions, while the North and South regions show lower absolute values. This pattern is consistent with previous mapping studies conducted by Lourenço et al. (1996) and Reckziegel and Pinto (2014), and more recently by Guerra-Duarte et al. (2023) and Lacerda et al. (2025).

In the Southeast region, the states of Minas Gerais and São Paulo stand out, presenting the highest cumulative numbers of cases, with 449,597 and 424,954 cases, respectively. This pattern may be associated with high levels of urbanization, population density, and environmental conditions favorable to scorpion proliferation, particularly in urban areas characterized by waste accumulation and inadequate sanitation infrastructure. In addition, the influence of meteorological variables and climate variability patterns, such as the El Niño–Southern Oscillation (ENSO), may contribute to these dynamics (Chiaravalloti-Neto et al., 2023). Furthermore, the adaptation of species such as *Tityus serrulatus* to urban environments plays a significant role in increasing incidence in this region.

In the Northeast region, states such as Bahia, Pernambuco, and Alagoas also exhibit high numbers of accidents, reinforcing the importance of climatic factors such as high temperatures and semi-arid conditions, combined with often unplanned urban expansion (Lisboa et al., 2020; Cavalcante Santos et al., 2023). These factors favor scorpion occurrence and dispersion, increasing the risk of accidents.

In contrast, states in the North region, such as Roraima, Acre, and Amapá, show the lowest recorded numbers of cases. However, this lower incidence should be interpreted with caution, as it may reflect not only distinct environmental conditions and lower population density but also potential limitations in epidemiological surveillance systems and underreporting.

Although the figure provides a comprehensive overview of the distribution of accidents, it is important to note that the data represent absolute values and are not adjusted for population size. As a result, more populous states tend to show higher numbers of cases, which may mask the true magnitude of the problem when compared proportionally. Therefore, analyses based on standardized incidence rates per 100,000 inhabitants are essential for a more accurate assessment of epidemiological risk.

In summary, the results indicate that scorpion sting accidents in Brazil are strongly concentrated in urbanized and climatically favorable areas, highlighting the need for public policies focused on environmental control, improved sanitation conditions, and strengthening of surveillance systems, particularly in the most affected regions.
